# Regional Variability in MRI Scans with Different Magnetic Field Strengths in Japan: Implications for Healthcare Preparedness for Alzheimer’s Disease Treatment

**DOI:** 10.3390/biomedicines12081870

**Published:** 2024-08-16

**Authors:** Kenichiro Sato, Yoshiki Niimi, Ryoko Ihara, Atsushi Iwata, Takeshi Iwatsubo

**Affiliations:** 1 Department of Neuropathology, Graduate School of Medicine, The University of Tokyo, Tokyo 113-8655, Japan; kenisatou-tky@umin.ac.jp; 2 Unit for Early and Exploratory Clinical Development, The University of Tokyo Hospital, Tokyo 113-8655, Japan; 3 Department of Healthcare Economics and Health Policy, Graduate School of Medicine, The University of Tokyo, Tokyo 113-8655, Japan; 4 Department of Neurology, Tokyo Metopolitan Institute for Geriatrics and Gerontology, Tokyo 173-0015, Japan

**Keywords:** Alzheimer’s disease, magnetic resonance imaging, amyloid-related imaging abnormalities, nationwide summary statistics, NDB open data, magnetic field strength

## Abstract

(1) Background: The 2023 approval of lecanemab for early-stage Alzheimer’s disease (AD) highlighted the need for routine 1.5T or 3.0T MRI scans to monitor amyloid-related imaging abnormalities (ARIAs). Regional disparities in MRI scan frequency, MRI scanner availability, and scanner magnetic field strengths could affect readiness for anti-amyloid therapy and lead to inconsistencies in ARIA detection nationwide. (2) Methods: We assessed regional variance in MRI scan frequency and field strength across Japan using the National Database (NDB) Open Data website, which summarizes Japanese public health insurance claims from the fiscal years (FYs) 2015 to 2021. We employed a mixed-effects model with prefecture-level random intercepts and slopes over time, subsequently categorizing prefectures into clusters based on MRI usage. (3) Results: 1.5T MRI was the most common magnetic field strength, remaining stable from FY2015 to FY2021. 3.0T MRI usage slightly increased, although the COVID-19 pandemic in FY2020 led to a maximum reduction of 5%. Prefecture-level variance was higher for 3.0T MRIs, with more frequent usage in western Japan. (4) Conclusions: This study highlights prefecture-level variance in MRI usage across Japan. The insights gained could be instrumental in improving healthcare preparedness for anti-amyloid treatment and patient management.

## 1. Introduction

Alzheimer’s disease (AD) is the leading cause of dementia [1], affecting an estimated 7 million people in the United States and 32 million people worldwide [2]. In Japan, approximately 5 million elderly individuals are estimated to be living with dementia [3]. Clinical manifestations of AD include impairments in memory, language, executive function, and visuospatial function.

Amyloid pathology, including amyloid plaque deposition in the brain, is the prerequisite for the pathological diagnosis of AD [1]; meanwhile, in clinical practice, AD has often been diagnosed based on its clinical symptoms, chronic disease course, and typical findings on brain MRI scans [4]. Therefore, sufficient performance in MRI imaging is important for the diagnosis of AD as well as for the diagnosis of other neurological diseases. In Japan, the 1.5 Tesla (T) magnetic field strength scanner is the most widely used type of MRI.

The United States and Japan’s approval of lecanemab (Leqembi™) for early-stage Alzheimer’s disease (AD) patients in 2023 highlighted the critical need for MRI monitoring to ensure safe administration, due to the risk of serious adverse effects known as amyloid-related imaging abnormalities (ARIAs) [5]. The Appropriate Use Recommendations (AUR) for lecanemab advise routine MRI scans with a minimum of 1.5 Tesla (T) for ARIA detection, with a preference for 3.0T scanners for enhanced sensitivity [6]. Similarly, Japan’s Optimal Use Guideline (OUG), issued in late 2023, requires routine MRI monitoring with a 1.5T minimum without specifying a preference between 1.5T or 3.0T scanners [7].

The 3.0T MRI scans, known for their superior signal-to-noise ratio, offer enhanced sensitivity to brain lesions [8], including both ARIA-edema/effusion (ARIA-E) and ARIA-hemorrhage (ARIA-H) [9]. However, the costs associated with high-field MRI scans (e.g., 3.0T) exceed those of 1.5T MRIs, and high-field MRI technology is not widely available. Consequently, universal 3.0T MRI monitoring for all patients on lecanemab may not be feasible.

A potential pitfall in the MRI monitoring of lecanemab treatment is the discrepancy in ARIA detection when patients undergo scans at varying magnetic field strengths. If a patient’s follow-up MRI scans alternate between 1.5T and 3.0T, it complicates the differentiation between new ARIA lesions and those previously undetected due to lower resolution. To mitigate this, consistency in the magnetic field strength used for MRI monitoring throughout a patient’s treatment is recommended to ensure accurate vigilance for ARIAs [9].

The introduction of lecanemab in Japan has heightened awareness regarding the Japanese healthcare system’s preparedness for lecanemab, including MRI monitoring capabilities [10,11,12]. Despite Japan boasting the highest per capita number of MRI machines globally [13], the majority of the used MRI scanners have 1.5T magnet, and scanners with lower fields (e.g., 1.0T) are still in active use. Although it has been known that the number of MRI scans undergone or the number of MRI scanners of any magnetic field strength vary by region (e.g., prefectures) [14], there may be further disparity in the proportion of the magnetic field strength of MRI scanners (e.g., 3.0T versus 1.5T). If so, it could impact regional readiness for anti-amyloid therapy, result in inconsistencies in ARIA detection rates across the country, and potentially introduce bias into the interpretation of the nationwide post-marketing surveillance of lecanemab. The same arguments about healthcare readiness associated with MRI usage can also be made not only in Japan, but also in other countries.

This study aims to quantitatively examine the degree of regional variations in the frequency and their field strength of MRI scans across Japan by analyzing data from the National Database (NDB) Open Data website (https://www.mhlw.go.jp/ndb/opendatasite/, accessed on 9 January 2024). We believe that the current study could serve as a model case for the study of MRI utilization.

## 2. Materials and Methods

### 2.1. Database

This was a retrospective observational study using the National Database of Health Insurance Claims and Specific Health Checkups of Japan (NDB Open Data) (https://www.mhlw.go.jp/ndb/opendatasite/, accessed on 9 January 2024) [15], which include the summary statistics of nationwide Japanese public health insurance claims in the fiscal years 2015 to 2021 and are publicly distributed by the Ministry of Health, Labour, and Welfare (MHLW) [16]. The fiscal year (FY) in Japan begins on April 1st and ends on March 31st of the next calendar year (e.g., FY2015: 1 April 2015~31 March 2016). The data were downloaded from the website on 9 January 2024, which required no permission or informed consent.

We used cumulative statistics on the frequency of MRI scans performed at outpatient clinics for each fiscal year, segmented by the prefecture (e.g., Tokyo, Osaka, Kyoto) and categorized by magnetic field strength: 1.5T [practice code: 170020110], 3.0T [practice code: 170033510 and 170035010], and lower field (e.g., <1.5T) [practice code: 170015210]. These are the aggregated counts of MRI scans across various imaging regions (e.g., brain, spine, joint, abdomen) from patients of all ages. Although the dataset does not provide detailed counts segregated by patient age or specific scan region beyond the prefectural breakdown, we presumed that the observed statistical trend might reflect the use of brain MRI scans among older individuals. This assumption is supported by the increase in nationwide MRI scan frequency along with the aging population, particularly from ages 20 to 74 years (Figure S1, modified from a screenshot of the NDB Open Data website: https://www.mhlw.go.jp/ndb/opendatasite/dai8kai/ikashinryou/sei_nennrei/index.html, accessed on 9 January 2024).

Furthermore, to account for regional variabilities that could influence the frequency of MRI scans, such as the size of the prefectural population or the degree of medical demand for brain disease diagnoses, we also sourced statistics on the prefectural population, the rate of death due to cerebrovascular diseases (e.g., the number of deaths per 100,000 population), and the count of MRI scanners in each prefecture for each fiscal year. These statistics are publicly available through the Japanese Government’s website (https://www.e-stat.go.jp/en, accessed on 9 January 2024). Since the count of MRI scanners was only surveyed in the fiscal years 2014, 2017, and 2021, the count of scanners in other fiscal years was interpolated using the R package {imputeTS} to estimate the data.

### 2.2. Analyses

All data preprocessing and statistical analyses were conducted using R software (R Foundation for Statistical Computing, Vienna, Austria. version 4.1.0) by one of our authors (K.S.). To assess the frequency of MRI scans across different magnetic field strengths (i.e., 1.5T, 3.0T, and less than 1.5T), which are expected to vary by prefecture, we employed a mixed-effects model with prefecture-level random intercepts and random slopes over time (i.e., FY2015 through FY2021), using R package {lme4} [17]. The negative binomial model equation, which estimates the annual frequency of MRI scans at different magnetic field strengths and by prefecture, is articulated as follows [18]:

(Model 1) $$\begin{array}{ll} ln\left(E\left(Y_{m,t,p}\right)\right)= & \beta_{0p}+\beta_{1p}T_{t}+\beta_{2p}M_{t,p}+\beta_{3p}T_{t}M_{t,p}+\beta_{4p}{FY2020}_{t}+offset\left(ln\left({Population}_{t,p}\right)\right)\\ & +\gamma_{1p}{MRI\left(1.5T\right)}_{t,p}+\gamma_{2p}T_{t}{MRI\left(1.5T\right)}_{t,p}+\gamma_{3p}{MRI\left(3.0T\right)}_{t,p}+\gamma_{4p}T_{t}{MRI\left(3.0T\right)}_{t,p}\\ & +\gamma_{5p}{MRI\left(1.5Tless\right)}_{t,p}+\gamma_{6p}T_{t}{MRI\left(1.5Tless\right)}_{t,p} \end{array}$$

In this model, *Y_m_*_,*t*,*p*_ represents the total annual count of MRI scans with magnetic field strength *m* in prefecture *p* at year *t*. *M_t_*_,*p*_ indicates the magnetic field type of the MRI scans (with 1.5T as the reference, 3.0T, and less than 1.5T) in prefecture *p* during year *t*. *T_t_* denotes the years elapsed since FY2015 (i.e., *t* = 0 in FY2015). *FY*2020_*t*_ is a binary indicator for FY2020 (i.e., equals 1 for FY2020 only, and 0 for other fiscal years) to account for the temporary impact of the COVID-19 pandemic on Japan’s healthcare system in early FY2020 [18]. The variables *MRI*(1.5*T*)*_t_*_,*p*_, *MRI*(3.0*T*)*_t_*_,*p*_, and *MRI*(1.5*Tless*)*_t_*_,*p*_ serve as dummy variables indicating whether the magnetic field strength of MRI scans is 1.5T, 3.0T, or less than 1.5T in prefecture *p* at year *t*.

The parameters *β*_0*p*_, *β*_1*p*_, *β*_2*p*_, and *β*_3*p*_ represent the fixed intercept, fixed slope, and additional fixed effects for MRI scans with 3.0T or less than 1.5T, respectively. *γ*_1*p*_, *γ*_3*p*_, and *γ*_5*p*_ are the prefecture-level random intercepts for MRI scans with 1.5T, 3.0T, and less than 1.5T, whereas *γ*_2*p*_, *γ*_4*p*_, and *γ*_6*p*_ denote the prefecture-level random slopes additional for each respective field strength. The exponentials of *β* and *γ* are interpreted as the incident rate ratio (IRR) for fixed effects and prefecture-level random effects.

For sensitivity analysis, we varied the offset term from prefecture-level population (*Population_t_*_,*p*_: model (1)) to the total annual number of deaths due to cerebrovascular diseases in prefecture *p* at year *t* (*Death_t_*_,*p*_: model (2)) or to the total annual number of MRI machines in prefecture *p* at year *t* (*Machine_t_*_,*p*_: model (3)). No harmonic term for incorporating seasonal fluctuations was included as the data are annual.

### 2.3. Prefectural Characterization

While the fixed effects in our models indicate the nationwide trend of MRI scans excluding the heterogeneity across prefectures, the random effects elucidate the prefecture-level variability. First, within the same model equation, we compared *γ*_1*p*_ with *γ*_3*p*_ and *γ*_2*p*_ with *γ*_4*p*_ in terms of their variability, utilizing the coefficient of variation (CV), which is calculated by the formula CV=SD/mean. This allows us to understand how the degree of prefecture-level variability in the frequency of MRI scans may differ between 1.5T and 3.0T scans. Comparison was achieved by examining whether the lower 95% of [*CV*_3.0*T*_ subtracted by *CV*_1.5*T*_] was higher than 0 in bootstrap (B = 1000).

Subsequently, we evaluated how individual prefectures have close or distant relationships with each other in terms of the serial frequency of MRI scans with different field strength. We converted time-series data into static features characterizing serial change in the frequency of MRI scans by random intercept, random slope, and the random effect at FY2021. We calculated the prefecture-level random effect at FY2021 in the model *m* (*m* = 1~3) by the following formulas:

$$\begin{array}{cc} G_{1.5T,\left(m\right)}=\gamma_{1p,\left(m\right)}+6\gamma_{2p,\left(m\right)} & Random\;effect\;for\;1.5T\;MRI\;scan\;at\;FY2021 \end{array}$$

$$\begin{array}{cc} G_{3.0T,\left(m\right)}=\gamma_{3p,\left(m\right)}+6\gamma_{4p,\left(m\right)} & Random\;effect\;for\;3.0T\;MRI\;scan\;at\;FY2021 \end{array}$$

$$\begin{array}{cc} G_{1.5Tless,\left(m\right)}=\gamma_{5p,\left(m\right)}+6\gamma_{6p,\left(m\right)} & Random\;effect\;for\;less\;than\;1.5T\;MRI\;scan\;at\;FY2021 \end{array}$$

Accordingly, we obtained a set of 9 variables (i.e., γ_1p,(m)_, γ_2p,(m)_, γ_3p,(m)_, γ_4p,(m)_, γ_5p,(m)_, γ_6p,(m)_, G_1.5T,(m)_, G_3.0T,(m)_, and G_1.5Tless,(m)_) for model *m* in prefecture *p*. We applied Uniform Manifold Approximation and Projection (UMAP) [19] for reducing 27 prefecture-level dimensions (=9 variables × 3 models) into two dimensions using the R package {*UMAP*}, followed by clustering the different prefectures by k-means. The optimal number of clusters was determined by the elbow method.

### 2.4. Ethics

This study was conducted in accordance with the ethical standards outlined in the 1964 Declaration of Helsinki and its amendments. This study received approval from the University of Tokyo, Graduate School of Medicine’s Institutional Ethics Committee (ID: 11628-(3)). No informed consent was required since this study only uses publicly available data.

## 3. Results

### 3.1. Overall Trends

**Figure 1:**
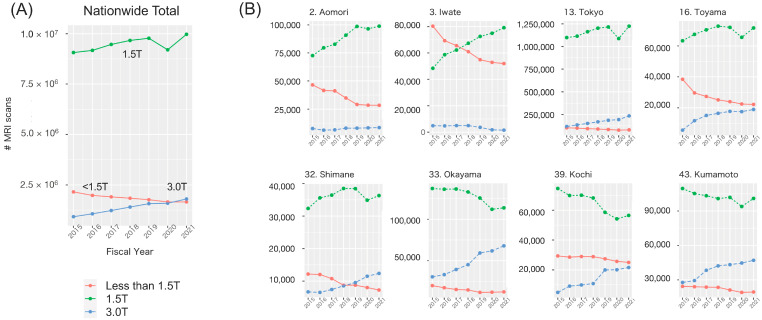
Serial trend of MRI scan frequencies. (**A**) Nationwide trend in Japan. (**B**) Characteristic trend in some prefectures.

The nationwide trends in MRI scan frequencies are depicted in Figure 1A. Generally, from FY2015 to FY2021, the annual count of 1.5T and 3.0T MRI scans gradually increased, while the annual count of MRI scans with a magnetic field strength of less than 1.5T steadily decreased. Specifically for 1.5T MRI scans, there appears to be a minor decline in frequency solely in FY2020, likely attributable to the COVID-19 pandemic.

While many prefectures exhibit trends consistent with the national aggregate, some prefectures display distinctive patterns (Figure 1B). For example, in *Aomori* (prefecture No. 2) and *Iwate* (prefecture No. 3), a shift in the predominant field strength from less than 1.5T to 1.5T is noted from FY2015 to FY2020, albeit with very limited availability of 3.0T MRI scans. Meanwhile, a suspected transition from lower-field to 3.0T MRI scans is suspected in *Toyama* (prefecture No. 16) and *Shimane* (prefecture No. 32). Additionally, a shift from 1.5T to 3.0T MRI scans is observed in *Okayama* (prefecture No. 33), *Kochi* (prefecture No. 39), and *Kumamoto* (prefecture No. 43). The trends of all 47 prefectures are illustrated in Figures S2–S4.

### 3.2. Mixed Model Results

The fixed effects from the mixed models are summarized in Table 1. The results across models (1)–(3) are largely consistent, corresponding to the characteristics suspected from the nationwide trend appearance in Figure 1A. The frequency of MRI scans with a magnetic field strength of 3.0T or lower than 1.5T (β_2_) is clearly less than that of 1.5T MRI scans, although its degree of decline varied by model. The impact of the COVID-19 pandemic in FY2020 (β_4_) resulted in an approximately 5% reduction, at best, in the frequency of MRI scans conducted. The overall trend from FY2015 to FY2021, as indicated by the slope (β_1_), largely remains stable over time, slightly varying by model. Additionally, the additional trend slope for 3.0T MRI scans (β_3_) shows a greater increase than that for 1.5T MRI scans.

The random intercepts for 3.0T MRI scans and for 1.5T MRI scans in models (1)–(3) are presented in Figure S5, and the random slopes for 3.0T MRI scans and for 1.5T MRI scans in models (1)–(3) are shown in Figure S6. In both models, the random intercept values for 3.0T MRI scans are largely distributed at a range of approximately 0.5–1.5, while a few prefectures had values as high as 2.5. The CV, as a measure of variance across prefectures, was higher for random intercepts of 3.0T MRI scans than for those of 1.5T MRI scans in all three models (e.g., a CV of 0.559 in Figure S5A compared to a CV of 0.197 in Figure S5C, and their difference was significantly higher than 0 in bootstrap).

### 3.3. Prefecture Characterization

**Figure 2:**
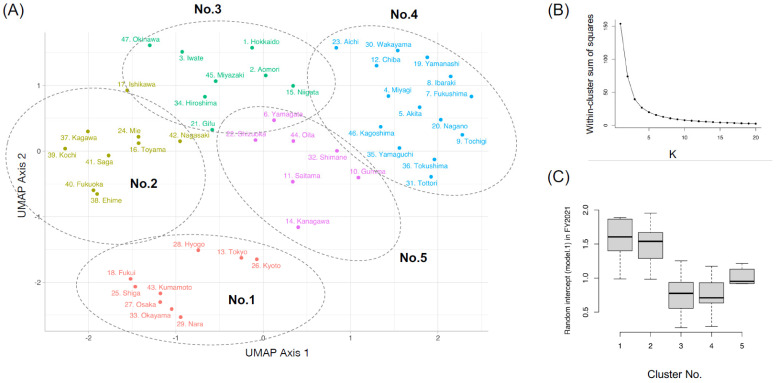
Clustering results of prefectures across Japan. (**A**) UMAP-based clustering of all prefectures. (**B**) Elbow method to determine the number of clusters in k-means. (**C**) Distribution of 3.0T MRI usage in five clusters.

The prefectures were categorized into five clusters (Figure 2A) based on the dimension reduction by UMAP on the 27 random effect variables and subsequent elbow method (Figure 2B). Prefecture clusters were numbered in an arbitrary manner. Prefectures within the same cluster have similar profiles to each other in terms of the frequency of MRI scans with different magnetic field strengths, and the inter-cluster distances in UMAP decomposition (Figure 2A) largely correspond to the degree of difference in the representative profile of MRI scans between prefectures within the clusters (Figure 2C).

**Figure 3:**
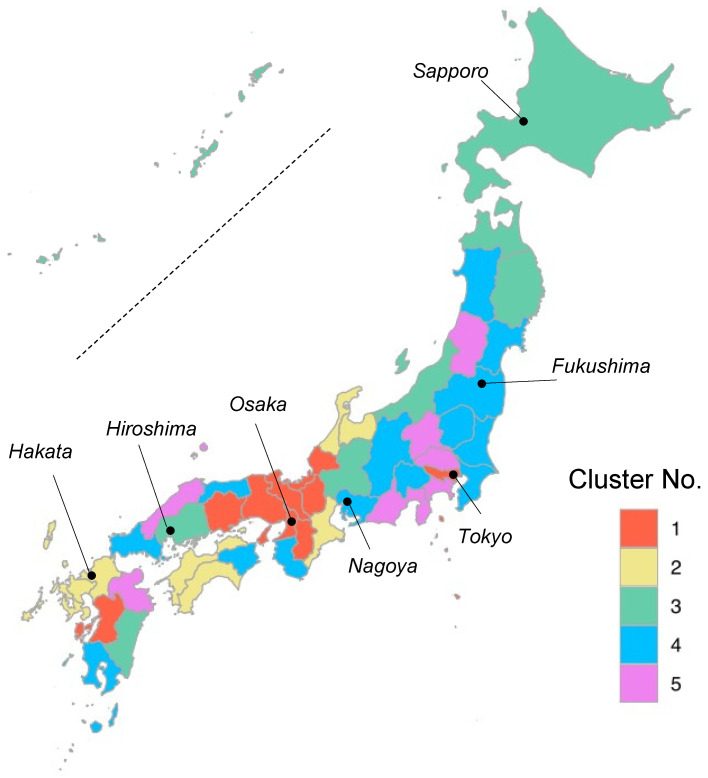
A choropleth map of prefectural clustering in Japan.

A choropleth map of the clustering is shown in Figure 3, roughly indicating that prefectures with a higher frequency of 3.0T MRI scans (e.g., cluster No. 1, 2, and 5) tend to be concentrated in western Japan.

## 4. Discussion

In this study, we quantitatively analyzed the degree of inter-prefecture variation in the frequency of MRI scans conducted with different magnetic field strengths. By applying a mixed-effect model, we were able to identify a serial MRI scanning trend that is common across prefectures and that varies by prefecture. In summary, 1.5T MRI was the magnetic field strength of the MRI scanners most predominantly used; the overall trend slope for 1.5T MRI scans from FY2015 to FY2021 largely remained relatively stable over time, and the trend slope for the 3.0T MRI scans exhibited a slight increase. The impact of the COVID-19 pandemic in FY2020 resulted in an approximately 5% reduction, at best. The prefecture-level variance, as represented by the random intercept, was found to be larger in 3.0T MRI scans than in 1.5T MRI scans. Furthermore, all 47 prefectures could be clustered into several groups based on the characteristics in their serial trend in MRI use. The current results examining the prefecture-level regional variance of MRI scans across Japan may prove useful in addressing challenges in healthcare preparedness for DMT treatment as well as in actual patient management.

Under Japan’s system of universal health coverage and uniform fees nationwide [20], it is postulated that the number of procedures per capita reflects the degree of available opportunities for taking 3.0T MRI scans for each person. This includes various factors such as the number of 3.0T MRI machines and their accessibility: e.g., conditions on their appointments or transportation to them. MRI scans are performed more frequently on the elderly individuals (Figure S1), and since the brain is one of the chief imaging areas, there might be a number of facilities that cannot perform MRI scans of the brain even with 3.0T scanners because of the absence of an MRI coil for the head and neck. This is why we assume that regional variability in the accessibility to 3.0T MRI, as one of the components of healthcare readiness for DMT provision, might have a substantial correlation with the variability in the actual frequency of 3.0T MRI scans. The same assumption cannot be applied to the NDB summary statistics of cerebrospinal fluid (CSF) test or PET scans [15], which are other components of healthcare readiness to DMT provision. This is because a non-negligible proportion of CSF testing has been conducted on children, and PET itself has been overwhelmingly used for cancer treatment.

Regional variance in the frequency of MRI scans or in the number of MRI scanners has already been acknowledged [14], and in this study, we did indeed observe variability in the random effects between prefectures (Figures S5 and S6). What is new in the current study is that we examined the regional variance separately according to the magnetic field strength. We also observed larger variability (i.e., CV) in the use of 3.0T scans than that in the use of 1.5T scans, which was the predominant field strength. This suggests that in future lecanemab treatment, the prefecture-level ARIA detection rate across Japan may potentially be influenced depending on the degree of use of 3.0T MRI. In Japan, a nationwide clinical registry was launched in early 2024 [21] to follow up patients treated with DMTs as a clinical investigation in addition to the post-marketing surveillance for DMT drugs (e.g., lecanemab); this registry is planned to collect safety information on ARIA cases and the MRI field strength used, so that the degree of influence of the field strength of MRI scans on the detection of ARIAs is expected to be validated in the future.

We categorized all 47 prefectures into five clusters, based on the characteristics in the serial change of use in MRI scans. The geographical distribution of the prefectural clusters shows that prefectures with a higher frequency of 3.0T MRI scans (e.g., clusters No. 1, 2, and 5) not only tend to be concentrated in western Japan, but also roughly correspond to the *Tokaido* corridor [22], the urbanization zone of Japan extending from the *Kanto* region (including *Tokyo*) through *Nagoya*, *Osaka*, and *Hiroshima* to the northern *Kyushu* region (including *Hakata*). In contrast, eastern or northern Japan (including *Fukushima* and *Sapporo*) may have relatively smaller levels of 3.0T MRI usage, although the reasons remain unclear.

From the clustering results, we can derive some helpful hints. For example, since prefectures belonging to the same cluster have similar profiles in the trend of use of MRI scans, there may be less concern about MRI monitoring for vigilance in ARIAs in patients receiving DMT treatment and changing to a new hospital for DMT treatment, along with moving out of one prefecture to another within the same cluster, mainly from the aspects of accessibility to MRI itself as well as the possible change in the magnetic field strength of the MRI scans to be received. Meanwhile, when patients have to move out to other prefectures outside of the previous cluster, discrepancies in the accessibility of MRI scans may be encountered depending on the degree of distance between the clusters.

Although the current study investigated the usage of MRI in Japan and its domestic distribution, the findings will also be applicable to other countries. For example, the same arguments about domestic regional discrepancies in MRI usage or magnetic field strength are also true for every country where lecanemab has been approved, especially for countries with a large area and intra-national life variability, such as the United States [6] or mainland China [23]. Similar analyses using appropriate nation-level statistics can also be considered in other countries, and we believe that the current study could serve as a model case for such analyses.

This study has some limitations. The current OUG in Japan strictly regulates the facilities where lecanemab treatment and MRI monitoring should be performed [7]. Our results, from determining prefectural clusters, are based on the MRI scans conducted at any hospital within each prefecture, but not on the MRI scans conducted at such lecanemab-available hospitals. Thus, our characterization of prefectures may actually differ from that of actual clinical practice in a strict sense. In addition, from the aspect of a geographically feasible range of hospital visits, a secondary medical care area, where general inpatient care and emergency care are established [24], may be a more important level of measurement of the degree of MRI utilization than the prefecture level, as was conducted in this study.

In the future, several additional research directions could be pursued; for example, we could conduct a comparative analysis of the performance of ARIA detection by MRI across countries, in terms of safety management and cost–benefit analysis. In addition, we could explore the feasibility and cost-effectiveness of possible health policy measures to improve the MRI infrastructure. Although separated from DMT treatment, a cost–benefit analysis should be conducted for the diagnosis and treatment of central nervous system disorders not limited to dementia. The importance of readiness for DMT treatment from the aspect of MRI infrastructure will become even more important, since donanemab, another anti-amyloid therapy drug for the treatment of early AD [25], was recently approved in July 2024 in the United States [26] and in August 2024 in Japan [27].

## 5. Conclusions

In conclusion, this study highlights the prefecture-level variance in MRI usage across Japan. The insights gained could be instrumental in improving healthcare preparedness for anti-amyloid treatment and patient management.

## Figures and Tables

**Table 1:** Fixed-effect results.

	Term	Variables		Coefficient	Lower 95%	Upper 95%
Model (1)	exp(β_0_)	(Intercept)		0.245	0.231	0.260
	exp(β_2_)	Field strength of MRI scanner	1.5T	Reference		
			<1.5T	0.252	0.211	0.302
			3.0T	0.099	0.082	0.119
	exp(β_1_)	Years since FY2015		1.000	0.993	1.006
	exp(β_4_)	Flag: FY2020		0.954	0.939	0.969
	exp(β_3_)	Interaction term	Field strength 1.5T × Years	Reference		
			Field strength < 1.5T × Years	0.950	0.937	0.963
			Field strength 3.0T × Years	1.107	1.077	1.138
Model (2)	exp(β_0_)	(Intercept)		66.433	60.457	73.000
	exp(β_2_)	Field strength of MRI scanner	1.5T	Reference		
			<1.5T	0.252	0.211	0.301
			3.0T	0.099	0.082	0.119
	exp(β_1_)	Years since FY2015		1.027	1.020	1.033
	exp(β_4_)	Flag: FY2020		0.982	0.965	0.998
	exp(β_3_)	Interaction term	Field strength 1.5T × Years	Reference		
			Field strength < 1.5T × Years	0.950	0.937	0.963
			Field strength 3.0T × Years	1.107	1.077	1.138
Model (3)	exp(β_0_)	(Intercept)		2048.127	1937.681	2164.868
	exp(β_2_)	Field strength of MRI scanner	1.5T	Reference		
			<1.5T	0.453	0.406	0.505
			3.0T	0.528	0.450	0.620
	exp(β_1_)	Years since FY2015		0.995	0.990	1.001
	exp(β_4_)	Flag: FY2020		0.951	0.937	0.966
	exp(β_3_)	Interaction term	Field strength 1.5T × Years	Reference		
			Field strength < 1.5T × Years	1.004	0.993	1.014
			Field strength 3.0T × Years	1.043	1.020	1.066

## Data Availability

The data supporting the findings of this study are openly available from the National Database of Health Insurance Claims and Specific Health Checkups of Japan (NDB Open Data) (https://www.mhlw.go.jp/ndb/opendatasite/, accessed on 9 January 2024).

## Supplementary Materials

The following supporting information can be downloaded at https://www.mdpi.com/article/10.3390/biomedicines12081870/s1: Figure S1: Nationwide MRI scan frequencies by age group; Figure S2: Serial trend of MRI scan frequencies for prefectures No. 1–16; Figure S3: Serial trend of MRI scan frequencies for prefectures No. 17–32; Figure S4: Serial trend of MRI scan frequencies for prefectures No. 33–47; Figure S5: Random intercept of each prefecture; Figure S6: Random slope of each prefecture.
