# Supplementary material for: Regional Variability in MRI Scans with Different Magnetic Field Strengths in Japan: Implications for Healthcare Preparedness for Alzheimer’s Disease Treatment
Source: Biomedicines. 2024 Aug 16;12(8):1870. doi: 10.3390/biomedicines12081870 (PMC11351322; doi:10.3390/biomedicines12081870)
Supplement: Supplementary file 1 [file biomedicines-12-01870-s001.zip › biomedicines-3088580-supplementary.pdf]

Supplementary Materials

Figure S1. Nationwide MRI scan frequencies by age group

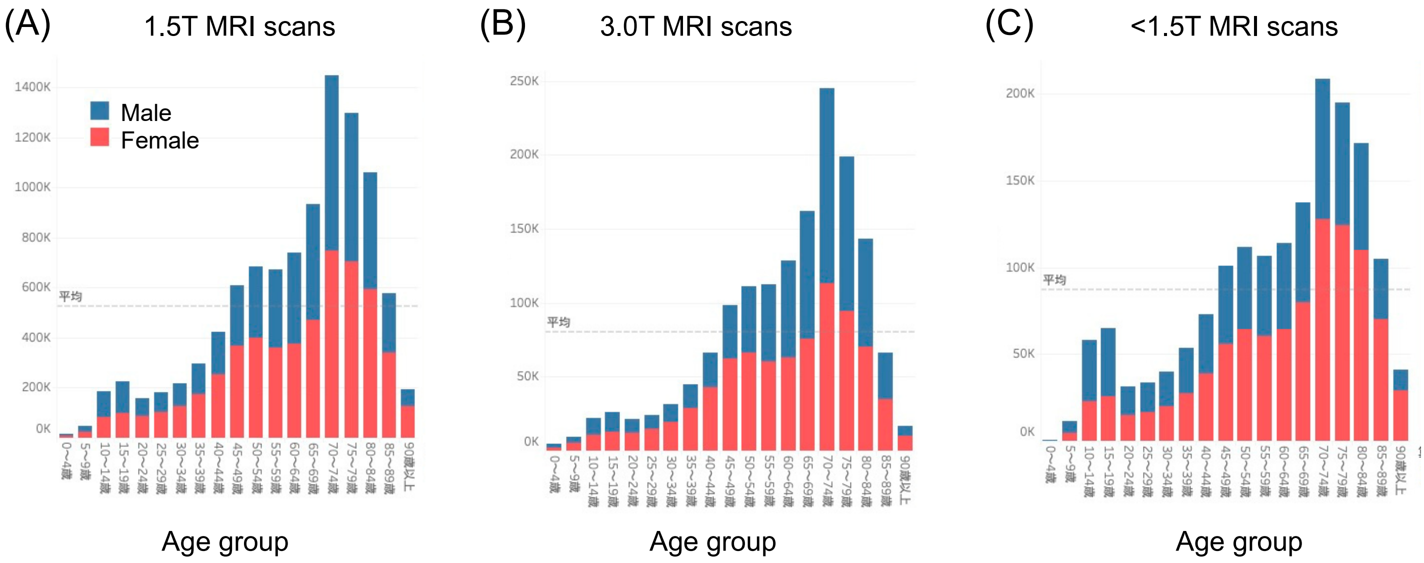

The frequency of nationwide MRI scans increase along with the aging, particularly from ages 20 to 74 years: modified screenshot from NDB Open Data website: https://www.mhlw.go.jp/ndb/opendatasite/dai8kai/ikashinryou/sei_nennrei/index.html).

Figure S2. Serial trend of MRI scan frequencies for prefectures No. 1-16

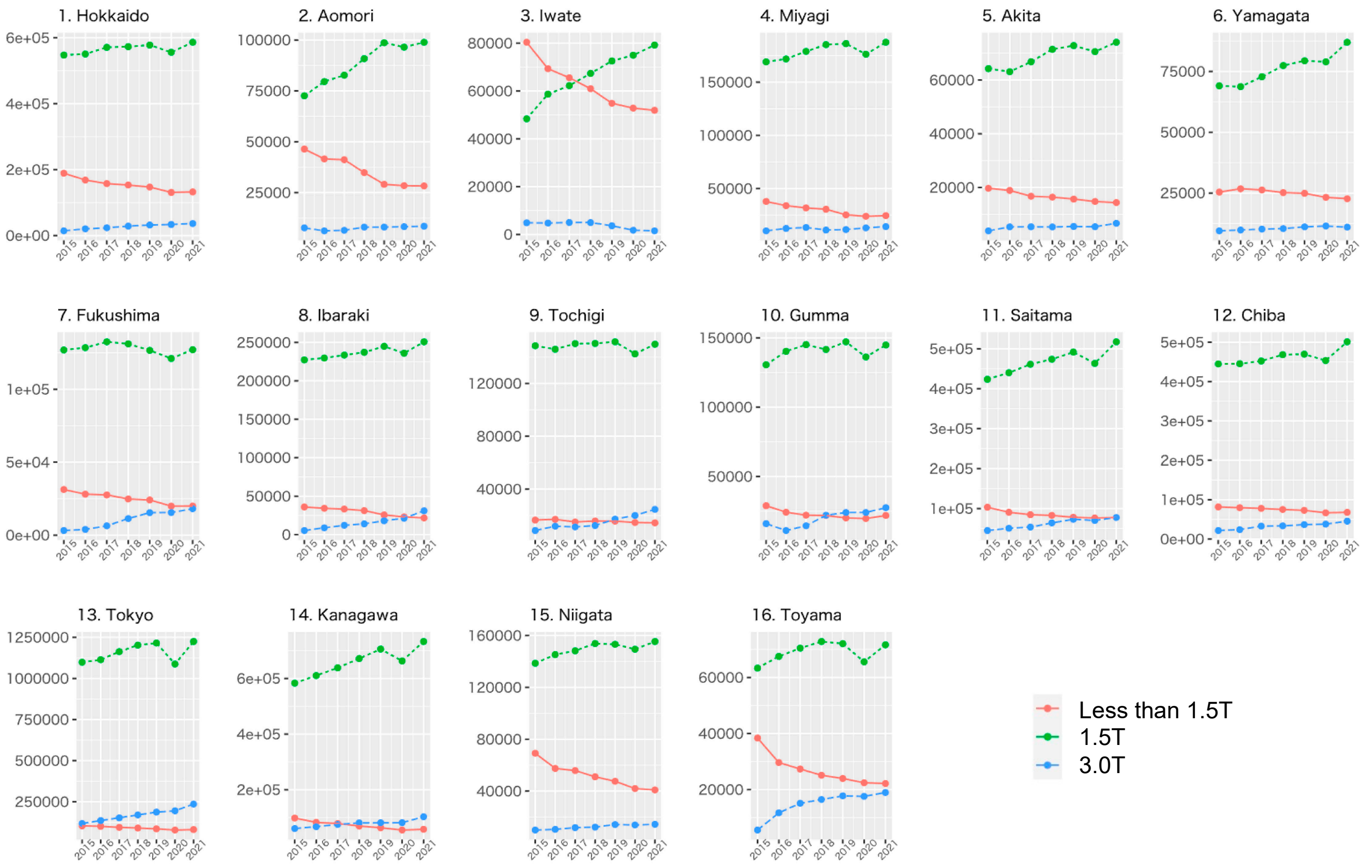

Abbreviations: T, tesla; MRI, magnetic resonance imaging.

Figure S3. Serial trend of MRI scan frequencies for prefectures No. 17-32

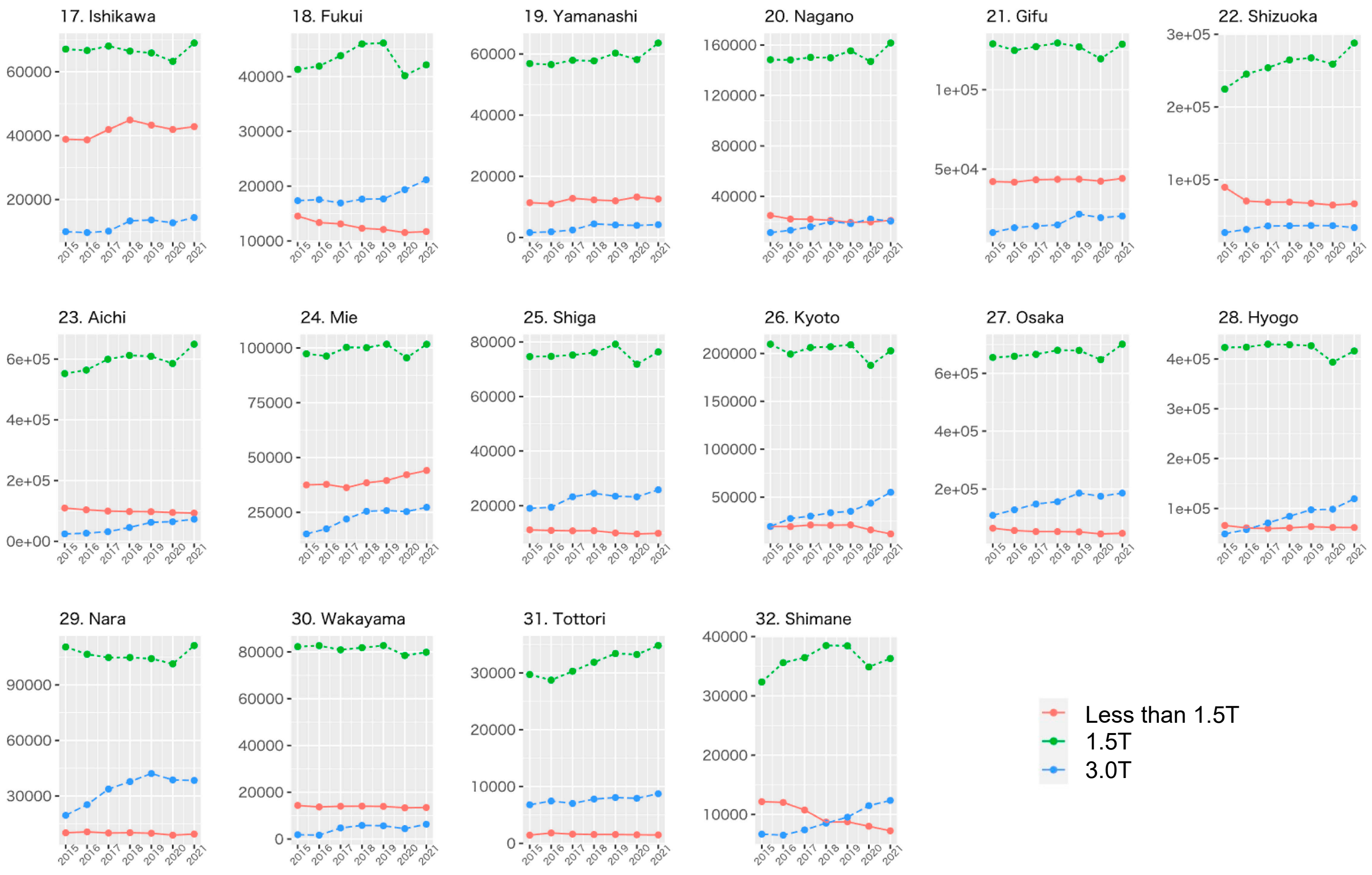

Abbreviations: T, tesla; MRI, magnetic resonance imaging.

Figure S4. Serial trend of MRI scan frequencies for prefectures No. 33-47

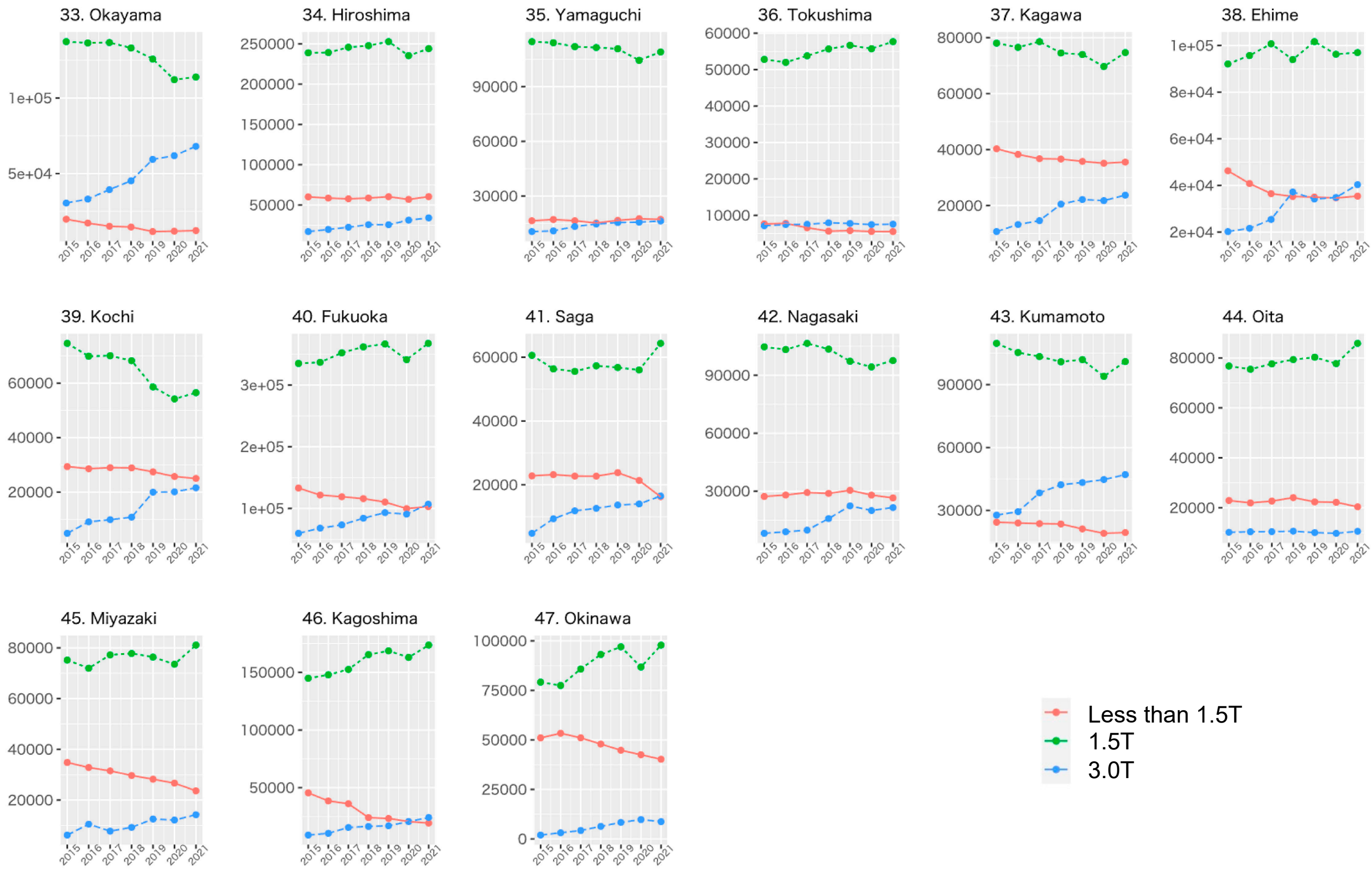

Abbreviations: T, tesla; MRI, magnetic resonance imaging.

Figure S5. Random intercept of each prefecture

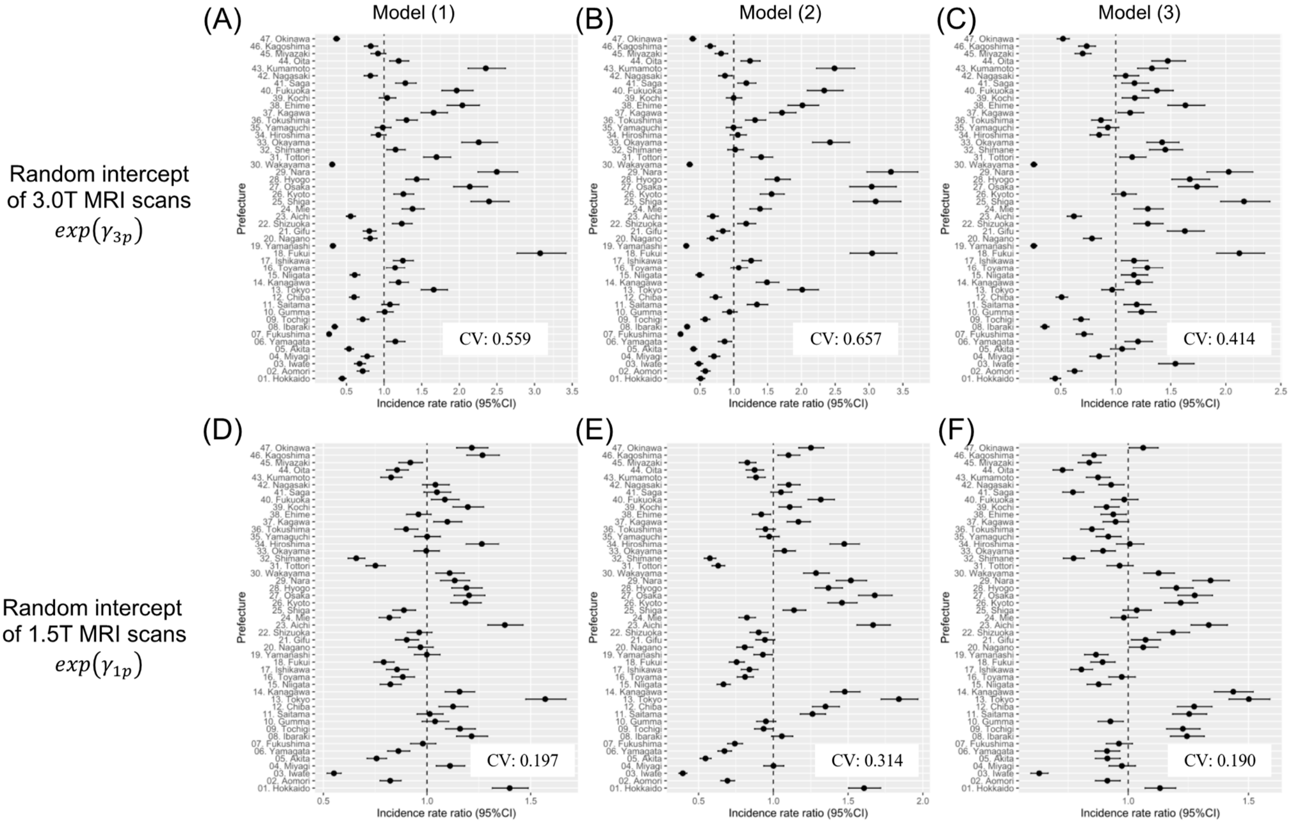

The random intercepts for 3.0T MRI scans and for 1.5T MRI scans in models (1)-(3) are presented in (A)-(C) and (D)-(F). In either models, random intercept values for 3.0T MRI scans largely distributed at a range of approximately 0.5-1.5, while a few of prefectures had a value as high as 2.5. The CV as a measure of variance across prefectures was higher for random intercepts of 3.0T MRI scans than for those of 1.5T MRI scans in all three models (e.g., CV of 0.559 in A compared to a CV of 0.197 in C, and their difference was significantly higher than 0 in boostrap).

Figure S6. Random slope of each prefecture

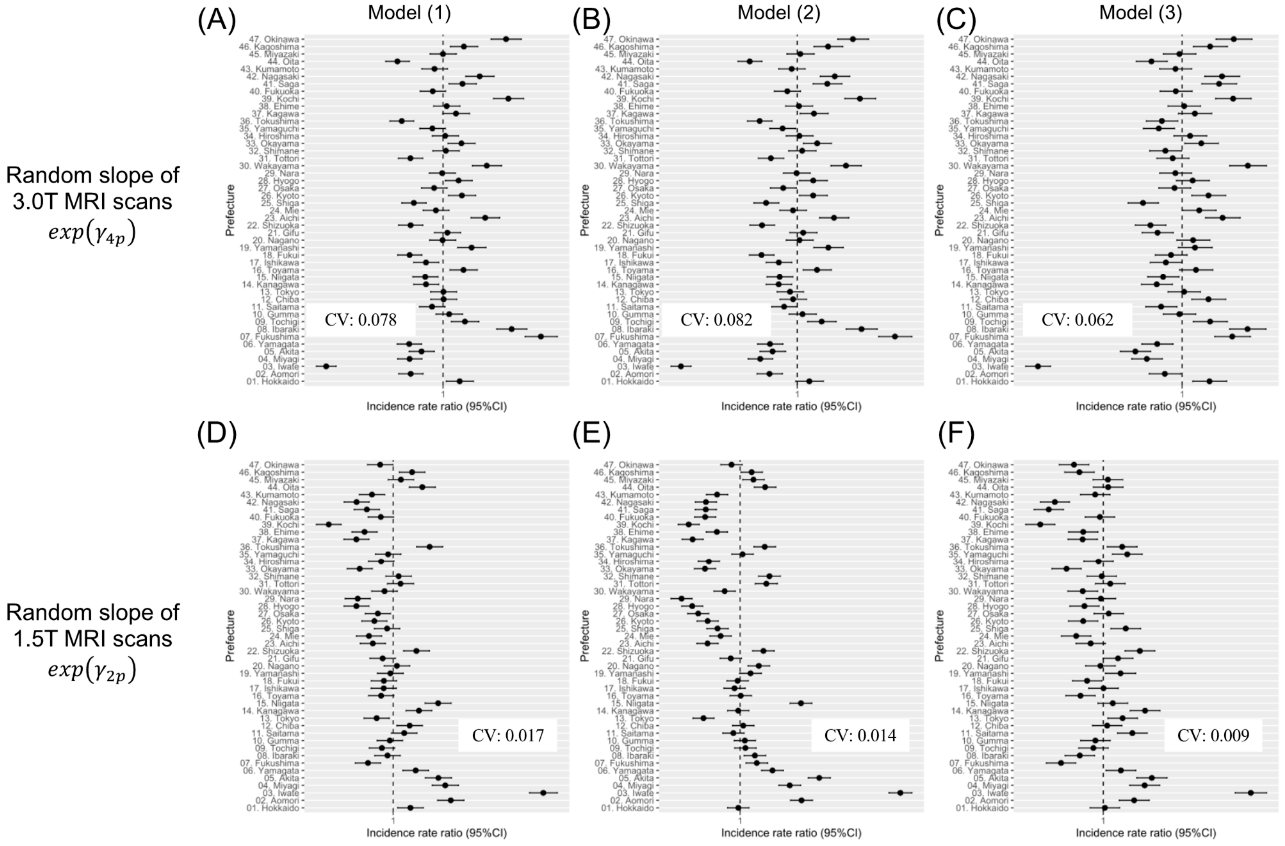

The random slopes for 3.0T MRI scans and for 1.5T MRI scans in models (1)-(3) are presented in (A)-(C) and (D)-(F).
